# Co-infection patterns in the ectoparasitic community affecting the Iberian ibex *Capra pyrenaica*

**DOI:** 10.1186/s13071-023-05797-y

**Published:** 2023-05-30

**Authors:** María J. Fernández-Muñoz, Raquel Castillo-Contreras, Jesús M. Pérez, José E. Granados, Francisco J. Márquez, Antonio J. López-Montoya

**Affiliations:** 1 Department of Animal and Plant Biology and Ecology, Universidad de Jaén, Campus Las Lagunillas, s.n., 23071 Jaén, Spain; 2 Fundación Artemisan, Av. Rey Santo 8, Portal Izquierdo, 2ª Planta, 13001 Ciudad Real, Spain; 3 Wildlife Ecology & Health Group (WE&H), Granada, Spain; 4 Sierra Nevada Natural Space, Carretera Antigua de Sierra Nevada, Km 7, 18071, Pinos Genil, Granada, Spain; 5 Department of Statistics and Operational Research, Universidad de Jaén, Campus Las Lagunillas, s.n., 23071 Jaén, Spain

**Keywords:** *Capra pyrenaica*, Co-infection, Ectoparasites, Epidemiology, Iberian Peninsula, Lice, *Sarcoptes scabiei*, Ticks

## Abstract

**Background:**

Sarcoptic mange is one of the main parasitic diseases affecting the Iberian ibex *Capra pyrenaica*. Scabietic animals suffer a decline in body condition and reproductive fitness and in severe cases may die. Although several previous studies of the pathology of this disease and the physiological changes it produces in ibex have been carried out in recent years, our knowledge of the relationship between *Sarcoptes scabiei* and other ectoparasites of this host is still limited.

**Methods:**

We analysed 430 Iberian ibex skin samples. Ectoparasites were removed, counted and identified. Mite (*S. scabiei*) numbers were obtained after digesting the skin samples in a 5% KOH solution. We modelled mite numbers in terms of host sex and age, site, year, season and the presence of other ectoparasites such as ticks and lice using generalized linear mixed models (GLMMs) and ectoparasite co-occurrence patterns using two different models: the probabilistic model species co-occurrence and the generalized linear latent variable model (GLLVM).

**Results:**

The ectoparasite community was mainly composed of *S. scabiei*, six ticks (*Haemaphysalis sulcata*, *Haemaphysalis punctata*, *Rhipicephalus bursa*, *Rhipicephalus turanicus*, *Dermacentor marginatus* and *Ixodes ricinus*) and two lice (*Bovicola crassipes* and *Linognathus stenopsis*). Adult male ibex harboured more mites than females. Mite numbers varied greatly spatially and seasonally and increased with the presence of other parasites. Some positive co-occurrence relationships between pairs of different ectoparasites were observed, particularly between ticks. The presence of *S. scabiei* negatively affected lice and *H. sulcata* numbers.

**Conclusions:**

Sarcoptic mange has spread above all in ibex populations in and around the Mediterranean Basin, where it is now found in almost a third of its host’s range. Mite numbers varied seasonally and spatially and were higher in male hosts. The presence of *S. scabiei* had a negative effect on lice numbers but favoured the presence of ticks.

**Graphical Abstract:**

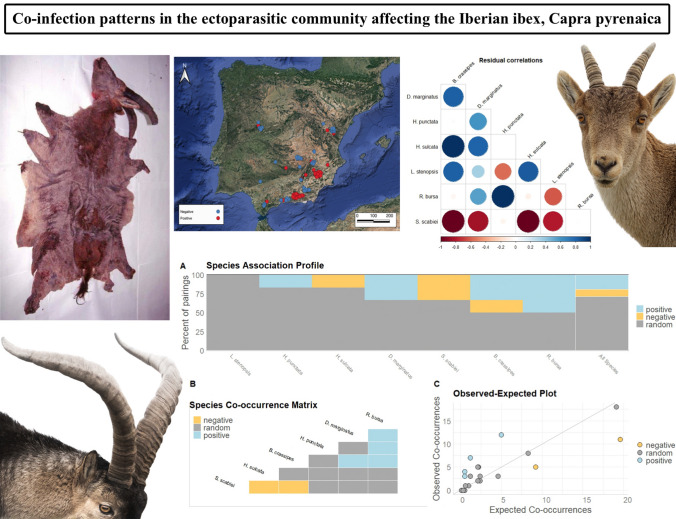

**Supplementary Information:**

The online version contains supplementary material available at 10.1186/s13071-023-05797-y.

## Background

Sarcoptic mange affects wild Caprinae throughout Eurasia [1–3]. In the Iberian ibex (*Capra pyrenaica*), outbreaks of this parasitic disease have been frequently recorded in the literature since the late 1980s, and much effort has since been dedicated to investigating the effects of sarcoptic mange at the individual and population levels in this particular host. As a consequence, certain aspects of the biology [4], ecology [5, 6], epidemiology [7, 8], physiology [9–12], pathology [13], genetics [14–16], diagnostic methods [17, 18] and management [19, 20] of this disease in this Caprinae species have only recently been explored. Briefly, a catabolic process leads ibex to lose weight [5, 10] and causes lesions on the skin and in inner organs (which are reversible), which are compounded by secondary infections [13] and a loss of reproductive fitness [9, 11]. After reaching its chronic phase, mange may kill hosts, although some ibex develop a degree of resistance [21, 22].

Hosts may become concurrently infected with several other micro- and macro-parasites [23], as occurs in Iberian ibex [24]. Parasites usually interact with each other, and these relationships may be antagonistic for at least one of the parasites or beneficial for one or both interacting parasites [25]. Pedersen and Fenton (2007) categorized a range of mechanisms that drive parasite interactions, ranging from reciprocal competition (i.e. for shared resources) to reciprocal facilitation (e.g. indirectly linked to the host’s immune response). Interactions between parasites may be similarly influenced by host traits such as behaviour, ecology, exposure history and pathologies [26] that affect the transmission [23, 27], distribution [28] and load patterns of parasites [29]. Morbidity induced by one parasite can affect host exposure to others, even if they are antagonistic [30], and mortality induced by one parasite can reduce the number of hosts available for other parasite species [31]. Moreover, the pattern of ectoparasite species co-occurrence varies over time and space [32].

Recently, Carvalho et al. [33] studied ectoparasite communities in ibex from the Sierra Nevada Natural Space (southern Spain). Such communities become richer more quickly in scabietic animals than in healthy ones. According to these authors, *Sarcoptes scabiei* infestations act in tandem with the off-host environment and host sex, which define the prevalence and abundance of lice and ticks. *Bovicola crassipes* was more prevalent in healthy animals, whereas *Linognathus stenopsis* was particularly prevalent in scabietic hosts with a severe clinical presentation.

The aims of this study were to (i) determine the occurrence of *S. scabiei* and other ectoparasites in ibex skin samples from different sites in Spain within the context of a monitoring programme of this disease; (ii) model the number of mites as a function of certain host and extrinsic factors; and (iii) analyse the co-occurrence patterns between these ectoparasites. We hypothesized the following: sarcoptic mange currently spreads through the Iberian Peninsula in parallel to host spread; epidemiology of sarcoptic mange in the Iberian Peninsula follows patterns found in intensively studied ibex population in Sierra Nevada (southern Spain); host alopecia caused by sarcoptic mange negatively affects lice (permanent ectoparasites attached to the host’s hair) but not ticks, which are temporal ectoparasites; the immune reaction caused by haematophagous parasites (e.g., ticks and sucking lice) may affect the presence of other ectoparasites.

## Methods

### Study area, sample collection and processing

**Fig. 1:**
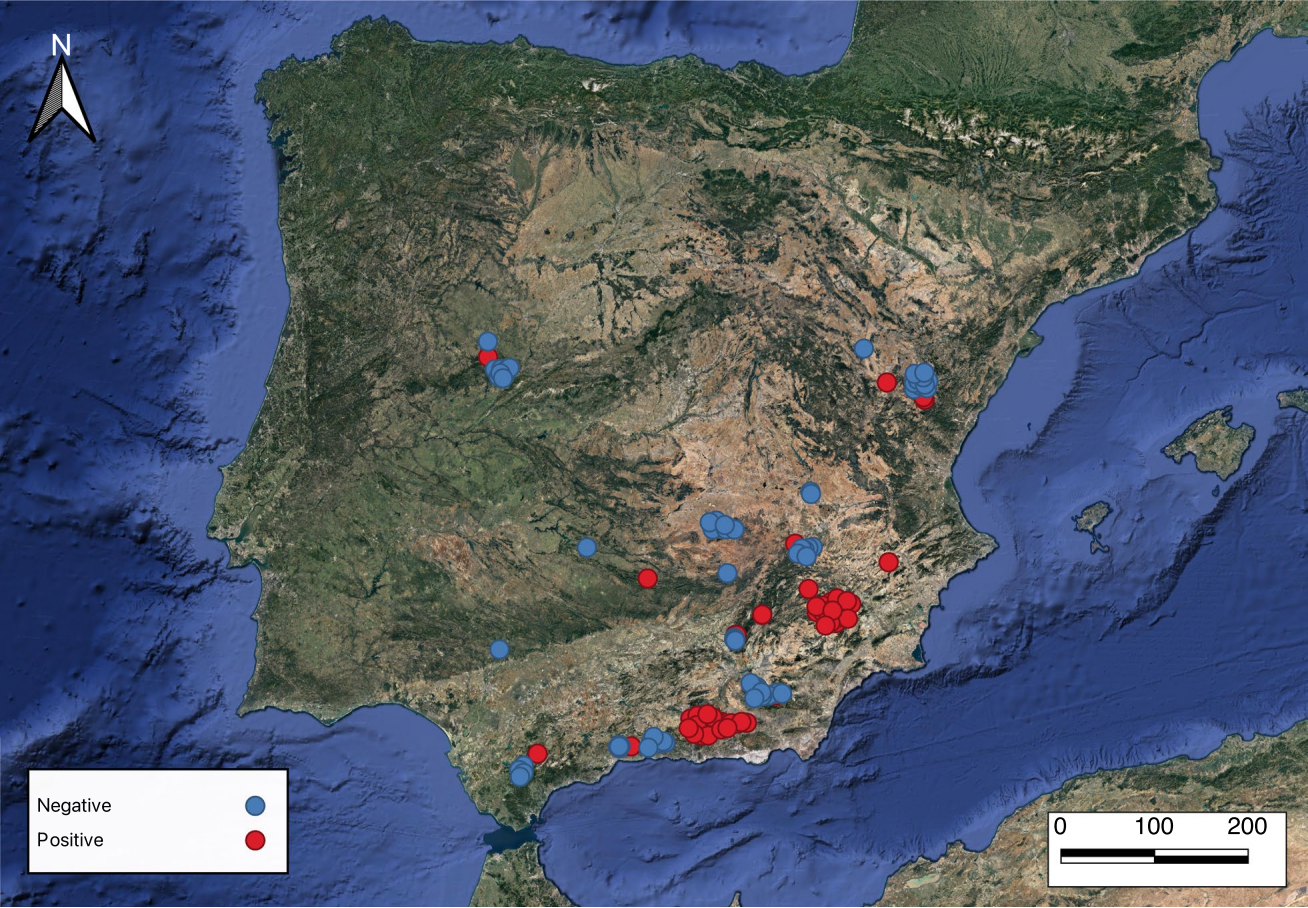
Geographical origin of the samples analysed in this study. Red dots: *Sarcoptes*-positive; blue dots: *Sarcoptes*-negative

In 2002–2022, 430 Iberian ibex skin samples (217 from males, 160 from females and 53 samples lacking information about sex) were provided by the staff of the Sierra Nevada Natural Space and the Fundación Artemisan (Ciudad Real, Spain). Samples were collected from legally hunted ibex harvested in Andalucía, Aragón, Castilla-La Mancha, Castilla-León and Región de Murcia (Fig. 1). Therefore, no approval by an ethics committee was necessary. A square 10 × 10-cm skin sample was removed from the withers of each shot animal, placed in a plastic bag, labelled and then frozen until analysis.

Each sample was inspected for ectoparasites, which were collected, counted and fixed in 70% ethanol. Lice and ticks were identified to species level using available morphological keys [5, 34–37]. A 2.5 × 2.5-cm portion of each skin sample was removed and digested in a 5% KOH solution overnight at 45 °C [4] and the number of mites was recorded.

### Statistical analysis

The database (*n* = 430) included 13 variables: ‘mite number’ or number of *Sarcoptes* specimens, host ‘sex’ and ‘age’, ‘site’ (where the host was shot), ‘year’ and ‘season’, when the sample was taken: ‘autumn’ (October–December), ‘winter’ (January–March), ‘spring’ (April–June) and ‘summer’ (July–September), and ‘others’ including the number of parasites other than *S. scabiei* on the host: two lice species (‘*B. crassipes’* and ‘*L. stenopsis’*) and five tick species (‘*Haemaphysalis sulcata*’, ‘*Haemaphysalis punctata*’, ‘*Rhipicephalus bursa*’, ‘*Rhipicephalus turanicus*’ and ‘*Dermacentor marginatus*’). *Ixodes ricinus* was not included in the analyses because it was present only (one adult female) in one host. Like the case of *Rhipicephalus turanicus* in which there are only three individuals.

All statistical analysis was carried out using R version 4.2.2. [38]. We also compared the number of *Sarcoptes* on Iberian ibex between seasons and sites separately for each year considered. Due to the lack of normality in the residuals of the ANOVA for both season and site, we used the Kruskal–Wallis test. This test was used to perform a comparison between the distributional form of the seasonal groups and site with certain simplifications; comparisons were carried out on the medians to detect significant differences in the number of mites between seasonal and site medians. A Dunn test was performed to check the multiple comparisons after the Kruskal–Wallis test. We used the kruskal.test() function to conduct the Kruskal–Wallis test with the *stats* package, while the Dunn test post hoc comparisons were estimated with dunnTest(). Generalized linear mixed models (GLMMs) were employed to determine whether sex, age, year, season and the presence of other parasites affected mite densities. We also considered the variable ‘site’ as a random factor in the model to avoid pseudoreplication [39]. The dependent variable (‘mite number’) had an excess of zeros, which required the use of zero-inflated distributions. Before fitting zero-inflated models, we carried out a zero-inflated test with the testZeroInflation() function in the *DHARMa* package in R [40], given that the presence of many zeros does not necessarily mean that there was a zero-inflation problem [41]. Zero-inflated Poisson and zero-inflated negative binomial mixed models were fitted using the glmmTMB() function of the *glmmTMB* package in R [42]. The conditional and marginal ${R}^{2}$ values based on [43] were obtained using the package *performance* [44]. There was no substantial correlation between explanatory variables when variation inflation factor (VIF) values were < 5 [45]. VIFs were obtained using the check_collinearity () function the of package *performance* [44]. We plotted the standardized estimates and random effects using the plot_model() function in the *sjPlot* package [46].

The first approach used to estimate the patterns of co-occurrence based on probabilistic model species co-occurrence [47] used the cooccur() function of the package *cooccur* [48]. This analysis uses a hypergeometric distribution to calculate the probabilities that a lower or higher value of co-occurrence may or may not be randomly obtained.

The second approach, the joint species distribution modelling framework, uses generalized linear latent variable models (GLLVMs) to assess how parasite community composition is influenced by environmental variation while taking into account patterns of species co-occurrence [49, 50]. We fitted GLLVMs using the gllvm() function of the *gllvm* package [51] in R, which incorporates the latent variables derived from the Laplace approximation method implemented through Template Model Builder [52]. The function gllvm() fits pure latent variable models (PLVMs) in which species occurrence data are regressed only against the latent variables [53].

Correlation between species co-occurrence could be due to residual correlation (e.g. unknown variables, biotic interactions, etc.), which can be accounted for by the latent variables in the PLVM [54]. The strength and sign of correlations between species co-infection were checked at a 5% significance level. A goodness-of-fit test was checked graphically with the summary() function of the *gllvm* package using a normal qq-plot of the residuals and the Dunn–Smyth residuals [55].

## Results

We identified eight ectoparasite species other than *S. scabiei* on the Iberian ibex skin samples: six tick species (*H. sulcata*, *H. punctata*, *R. bursa*, *R. turanicus*, *D. marginatus* and *I. ricinus*), together with a biting louse (*B. crassipes*) and a sucking louse (*L. stenopsis*). Table 1 summarizes their taxonomy, feeding habits and temporal relationship with their hosts.

**Table 1:** Relationship between ectoparasites found in this study, including their taxonomic group (at the family level), feeding habits and the temporality of their relationship with hosts

Parasite	Taxonomic group	Feeding habits	Temporal relation
*Sarcoptes scabiei*	Acari: Sarcoptidae	Microphagous	Permanent
*Haemaphysalis sulcata*	Acari: Ixodidae	Haematophagous	Temporal
*H. punctata*	Acari: Ixodidae	Haematophagous	Temporal
*Rhipicephalus bursa*	Acari: Ixodidae	Haematophagous	Temporal
*R. turanicus*	Acari: Ixodidae	Haematophagous	Temporal
*Dermacentor marginatus*	Acari: Ixodidae	Haematophagous	Temporal
*Ixodes ricinus*	Acari: Ixodidae	Haematophagous	Temporal
*Bovicola crassipes*	Phthiraptera: Trichodectidae	Dermal debris	Permanent
*Linognathus stenopsis*	Phthiraptera: Linognathidae	Haematophagous	Permanent

*Sarcoptes scabiei* was the most prevalent ectoparasite, affecting more than 46% of sampled animals. Nevertheless, the prevalence of mange varied significantly according to host origin: < 5% in Málaga and Salamanca provinces to > 50% in Granada, Jaén and Murcia provinces. *Rhipicephalus bursa* was found in almost 11% of sampled hosts and was the most abundant tick species. *Bovicola crassipes* was the most prevalent lice species, although *L. stenopsis* was the most abundant. The prevalence and mean intensity (± standard deviation) of each ectoparasite are shown in Table 2.

**Table 2:** Basic epidemiological data of the ectoparasites found on Iberian ibex

Parasite	Positive cases	Prevalence (%)	Mean intensity ± SD
*Sarcoptes scabiei*	200	46.5	54.4 ± 71.7
*Haemaphysalis sulcata*	22	5.1	3.1 ± 2.2
*H. punctata*	11	2.6	4.4 ± 5.1
*Rhipicephalus bursa*	46	10.7	7.5 ± 7.6
*R. turanicus*	3	0.7	2.0 ± 1.7
*Dermacentor marginatus*	5	1.2	5.8 ± 6.6
*Ixodes ricinus*	1	0.2	1 ± 0.0
*Bovicola crassipes*	47	10.9	4.6 ± 9.7
*Linognathus stenopsis*	20	4.7	13.9 ± 33.8

*SD* standard deviation

**Fig. 2:**
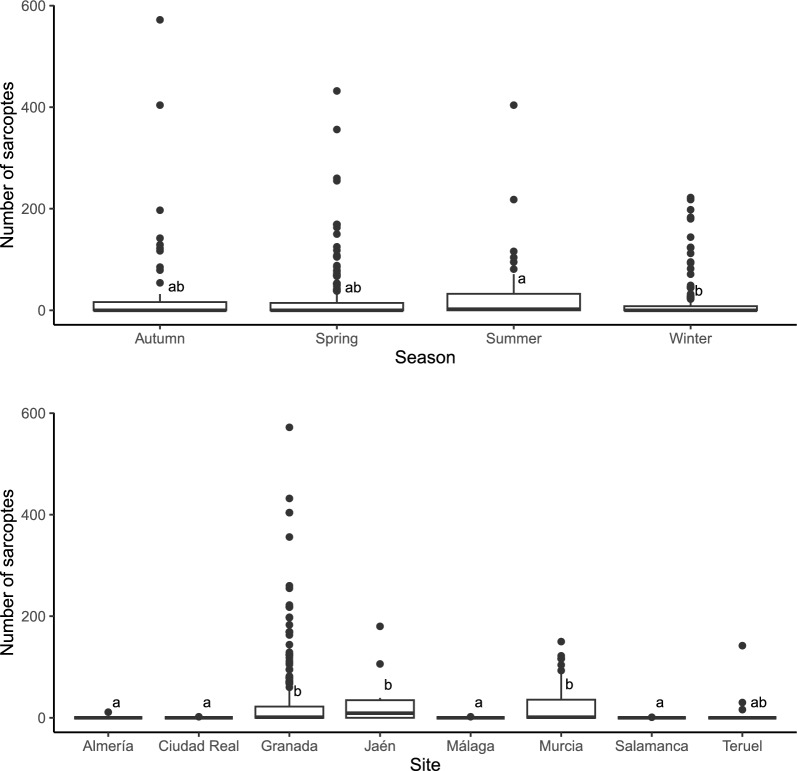
Variation of mite numbers in different seasons and sites from which skin samples originated

The Kruskal–Wallis test (Fig. 2) detected significant differences between seasons (${\chi }^{2}$=9.5233, *P*-value = 0.0231) and sites (${\chi }^{2}$=59.723, *P*-value < 0.0001). Post hoc multiple comparisons with the Dunn test showed statistically significant differences. The top plot shows differences between winter and summer, while the bottom plot shows differences between Granada, Jaén and Murcia and the other localities.

**Fig. 3:**
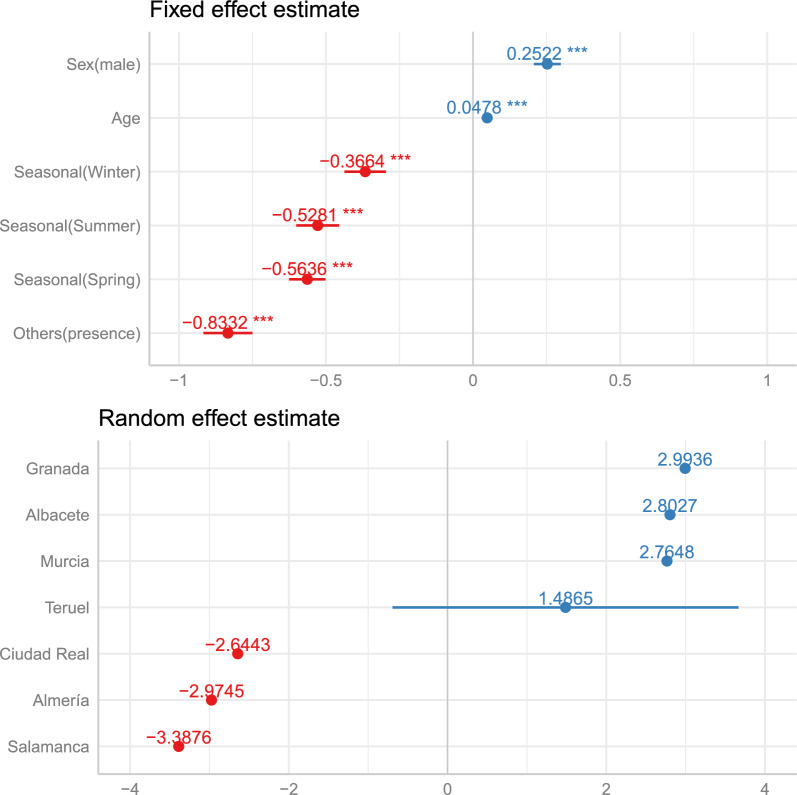
Influence of fixed and random factors on the number of *Sarcoptes* mites in Iberian ibex (*Capra pyrenaica*); estimated coefficients (and 95% confidence intervals) of the covariates for the explanatory variables; overlapping 95% confidence intervals 0 (solid vertical line) indicate a non-significant coefficient

**Fig. 4:**
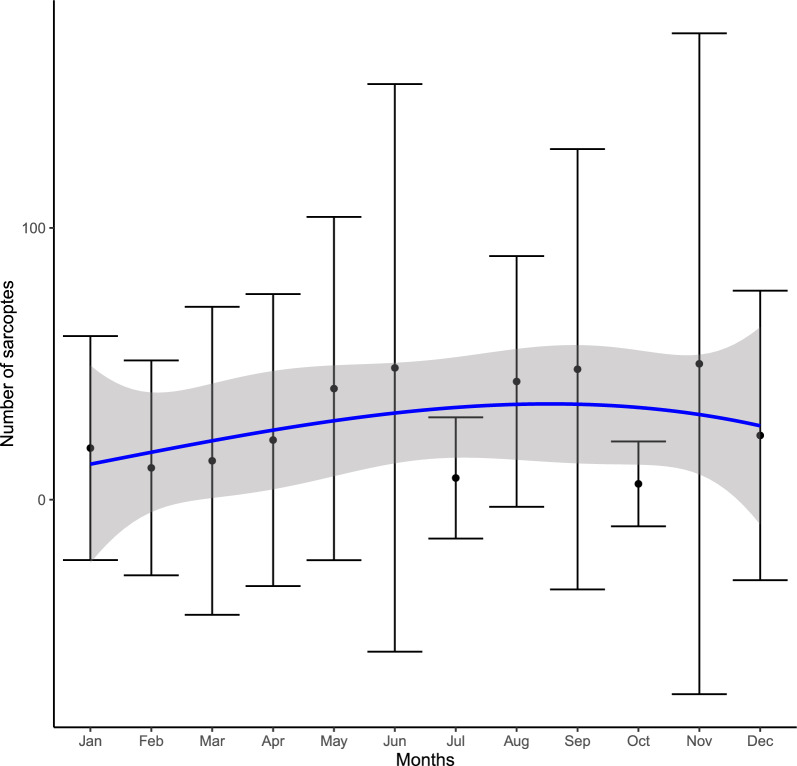
Monthly dynamics of *Sarcoptes scabiei* numbers. Points refer to the monthly mean value for mite numbers; bars represent the standard deviation; the blue line represents the smoothed average values; the grey area is the associated 95% confidence intervals

Goodness of fit of GLMMs and PLVMs was checked graphically (see Additional file 1: Figs. S1, S2 and S3) and we found that a zero-inflated Poisson distribution fitted better than the zero-inflated negative binomial distribution in the GLMMs. The zero-inflation test gave a ratioObsSim value of 2.0194, where a value of ratioObsSim > 1 means that there are more zeros than expected (also known as zero-inflation), as in our case. The zero-inflated Poisson GLMM found significant differences for all the parameters considered in the model and all *P*-values were below the 5% significance level (i.e. sex, age, year, season and others; see Table 3 and Fig. 3). VIF values were all < 3 for all the explanatory variables in the GLMM (Additional file 1: Table S1), so our model did not have multicollinearity problems. According to the coefficients of the model, autumn was the season with the highest number of mites on hosts (Fig. 4), males (particularly older ones) harboured more mites than females, and the presence of other parasites (ticks and/or lice) was negatively affected by the number of mites on ibex (see Table 3 and Fig. 3). Figure 3 depicts the random effects by levels.

**Table 3:** Summary of the results of estimates for zero-inflated Poisson GLMM models

Fixed effects	Estimate	SE	*z*-value	*P*-value
Intercept	2.6302	0.8472	3.105	0.0019
Sex (male)	0.2522	0.0233	10.847	< 0.0001
Age	0.0478	0.0049	9.835	< 0.0001
Spring	−0.5636	0.0315	−17.897	< 0.0001
Summer	−0.5281	0.0371	−14.219	< 0.0001
Winter	−0.3664	0.0359	−10.197	< 0.0001
Others (presence)	−0.8332	0.0425	−19.602	< 0.0001

Zero-inflated model	Estimate	SE	*z*-value	*P*-value
ZI-intercept	−0.0157	0.1149	−0.137	0.891
Random effects				
${\sigma }^{2}$	1.19			
${\tau }_{00 Site}$	5.76			
ICC	0.87			
${N}_{Site}$	7			
Marginal ${R}^{2}$/Conditional ${R}^{2}$	0.022/0.872			

From left to right: Parameter of the predictor variable, parameter estimate, estimate standard error (SE), *z*-value and *P*-value. In the parameter column, the ZI-intercept is the intercept of the zero-inflated part of the model. In the random effects column, ${\tau }_{00 Site}$ is the group variance, ${\sigma }^{2}$ the residual variance, ICC the intraclass correlation coefficient, and ${N}_{Site}$ the levels of the random factor. Autumn, female and no presence of ticks and/or lice are the baseline categories for the categorical explanatory variables

**Fig. 5:**
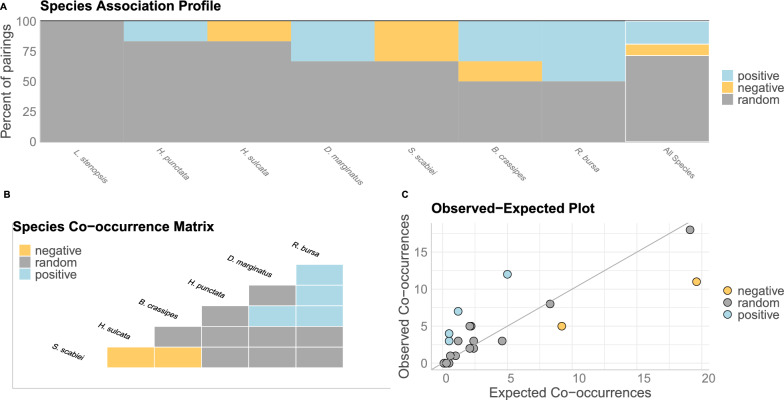
**A** The percent of total positive, negative or random pairings for each species. **B** Heat map representing the positive, negative or random species associations (co-occurrence). **C** Scatter plot with the observed vs expected co-occurrence. Positive, negative and random pairs of species are represented by coloured points

The co-occurrence analysis carried out by the methodology implemented in the *cooccur* package found six pairs of combinations. Figure 5A shows the percentage of species pairs that were classified as positive, negative or random for all species, and also illustrates whether the species tended to have predominantly positive or negative interactions. Additionally, this graph shows whether these interactions were uniformly distributed, since the bars are arranged in increasing (or decreasing) order. We found four positive, two negative and nine random or undefined associations (Fig. 5B); only significant associations are shown and events without co-occurrence data were removed. The positive associations were *R. bursa*–*D. marginatus*, *R. bursa*–*H. punctata*, *R. bursa*–*B. crassipes* and *D. marginatus*–*B. crassipes*, while the negative associations were *S. scabiei*–*H. sulcata* and *S. scabiei*–*B. crassipes*. The rest of the associations were classified as random (Fig. 5B). Figure 5C shows the observed and expected values of the co-occurrences and the degree to which the pairs of parasite species deviate from their expected levels of co-occurrence.

**Fig. 6:**
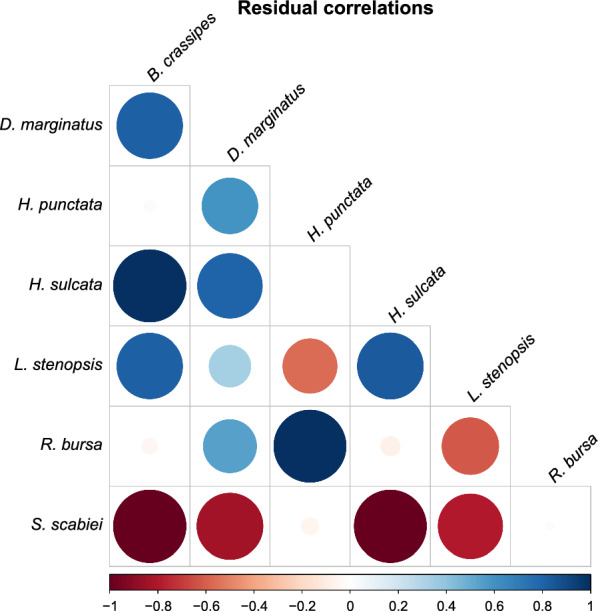
Correlations between parasite abundance due to latent variables based on the PLVM. The strength and significance of correlations (i.e. 95% confidence interval not overlapping zero) are represented by solid colours, while transparent ones represent non-significant correlations

The co-occurrence analysis carried out with PLVM shows that the patterns of co-occurrence between parasite species could be attributed to the effects produced between the parasites themselves. Figure 6 shows the correlations between the parasite species. We found the same associations as with the previous methodology, as well as six fresh ones. The positive associations were *B. crassipes*–*H. sulcata*, *D. marginatus*–*H. sulcata*, *B. crassipes*–*L. stenopsis* and *L. stenopsis*–*H. sulcata*, while the negative associations were *S. scabiei*–*D. marginatus* and *S. scabiei*–*L. stenopsis*.

In short, both probabilistic models and PLVM suggest that the patterns of co-occurrence between the six ectoparasite species can be attributed to the effects produced by the parasites themselves. Most of the significant correlations between the different ectoparasite pairs were positive—for example, those between the ticks; on the other hand, the presence of *S. scabiei* negatively affected the number of individuals of the lice species and of *D. marginatus* and *H. sulcata* (Figs. 5 and 6).

## Discussion

Our sampling method (including data on ectoparasites from 10 cm^2^ skin samples from host withers), despite being standardized, may represent a limitation of this study, as ectoparasites may be unevenly distributed over the skin surface. In fact, this may explain the large number of zeroes in our database.

Other arthropod species such as *Dermacentor reticulatus*, *Hyalomma lusitanicum*, *Psoroptes* sp., *Trombicula* sp. [56], *Straelensia cynotis* [57] and *Pulex irritans* [58] have been reported to parasitize Iberian ibex. These taxa were not included in our analyses due to their very low prevalence (only one or a very few cases) and lack of data on mite numbers, as they were found in other research projects.

Geographically, sarcoptic mange mainly affects the ibex populations in the Mediterranean Basin but does reach the north-west of the Iberian Peninsula (Riaño, Castilla-León) as well. Currently, this disease is present in over 28% of the distribution range of *C. pyrenaica* [59]. It spread throughout the whole of the Sierra Nevada mountain range in the 10 years following the detection of the first cases (1992), with an estimated mean front spread speed of nearly 9 km/year [60]. Moreover, given that the Iberian ibex is currently expanding its range [61], a similar trend in the future distribution of mange is to be expected.

The prevalence values obtained for the different host locations (provinces) must be interpreted with caution since most samples were not obtained randomly and mangy animals were more likely to be selectively removed in different areas for humanitarian reasons and/or to manage ibex density and mange spread. In fact, a decreasing trend in mange prevalence has recently been reported in the ibex population from Sierra Nevada [22] despite the fact that more than 58% of samples from this location were positive for *S. scabiei*.

The epidemiological trend observed in our study fits that previously reported for the Sierra Nevada Natura Space (southern Spain) [4]$.$ As expected, male ibex harboured more mites than females. This is due to physiological differences between the sexes, in particular in relation to the activity of sex steroid hormones such as testosterone, which has an immunosuppressive effect [4, 6]. Seasonal dynamics of mite numbers seem to be related to the concentrations of these hormones, with higher mite numbers—particularly larvae—coinciding with the host rutting season [62].

Nakagawa’s conditional ${R}^{2}$ for the selected zero-inflated Poisson GLMM explained 87.2% of the variance in the number of mites (Table 3). Information regarding other factors such as host body weight, kidney fat index (KFI) [4], immune response [8, 63] and temperature and humidity [64], among others, could improve this model in future.

Community resilience closely depends on the nature and strength of interspecific interactions [65]. The predominant pattern of species association within a community will determine the pattern of the community structure such that, for example, if most species associations are positive, the community will be structured aggregatively, with the frequency of species co-occurrence being greater than expected under random species assemblage. However, if these associations are negative, then the community structure is segregative, with the frequency of species co-occurrence being smaller than expected under random assemblage [66].

It is likely that competitive interactions between ticks and other haematophagous ectoparasites will occur due to competition for blood as a food resource [29]. Nevertheless, in our case, most of the significant interspecific associations between ibex ectoparasites were positive, so the community structure is aggregative and stable [33]. Aggregative patterns such as those shown by most of the tick species in our study suggest apparent facilitation mediated by the host. This facilitation could be explained by host immunodepression due to infection by multiple parasites [23, 67]. Establishing different types of immune responses is likely to be more costly than developing just one specific type of response [68]. Consequently, the effectiveness of energy allocation to immune defence will decrease as the diversity of parasite attacks increases [69]. Tick feeding induces a complex immune response in hosts [70]. Competition between tick species could also be reduced by temporal differences in emergence and/or attachment to hosts as a kind of segregation [71]. Co-occurrence between different lice taxa has not often been reported [72]. In our case, *B. crassipes* and *L. stenopsis* do not compete for food since their diet is quite different (Table 1). Again, the immune response developed by the host due to the haematophagous nature of *L. stenopsis* could facilitate the presence of *B. crassipes*.

As expected, the presence of *S. scabiei* negatively affected the presence of both lice species [33]. Mange induces alopecia in hosts, thereby reducing the ability of lice to remain attached to hosts. Nevertheless, these mites were also negatively associated with *D. marginatus* and *H. sulcata*, which suggests a cross-effective immune response in hosts [25].

As ibex are also infected by endoparasites, most of the possible interactions throughout the whole parasite community remain unexplored, which constitutes a challenge for future research.

## Conclusions

Our data evidence that sarcoptic mange is spreading across the Iberian Peninsula, parallel to host dispersal, as had been hypothesized. As previously reported for the Sierra Nevada ibex population, the number of mites and therefore the effects of this disease are biased toward host males and have a clear seasonal pattern; therefore, our starting hypothesis is also confirmed. *Sarcoptes scabiei*, together with five tick and two lice species, form a stable ectoparasite community in which the presence of mites usually favours the presence of ticks but constrains lice numbers, confirming our hypothesis in this regard. Some authors suggest performing manipulative experiments (e.g., involving the extirpation of one ectoparasite species) to confirm the reliability of such interspecific associations between ectoparasites) [33]. Nevertheless, such experiments are logistically challenging, as they need completely specific methods for removing a particular ectoparasitic species with no effects for the remaining ones. Further studies will allow us to assess potential co-infection patterns between *S. scabiei* and ibex endoparasites.

## Supplementary Information

**Additional file 1: Table S1.** Variation inflation factors (VIFs) with the zero-inflated Poisson GLMMs. **Figure S1.** Diagnostic plot of the residuals of the zero-inflated Poisson GLMM. **Figure S2.** Diagnostic plot of the residuals of the zero-inflated negative binomial GLLVM. **Figure S3.** Diagnostic plot of the residuals of the zero-inflated Poisson GLLVM.

## Data Availability

Data are available from the authors upon reasonable request and with permission from the Fundación Artemisan.
