# Supplementary material for: Co-infection patterns in the ectoparasitic community affecting the Iberian ibex Capra pyrenaica
Source: Parasit Vectors. 2023 May 30;16:172. doi: 10.1186/s13071-023-05797-y (PMC10228096; doi:10.1186/s13071-023-05797-y)

Table S1: Variation inflation factors (VIFs) with the zero-inflated Poisson GLMMs.

| Term | VIF | VIF 95% CI | Increased SE | Tolerance | Tolerance 95% CI |
| --- | --- | --- | --- | --- | --- |
| Sex | 1.06 | [1.01, 1.39] | 1.03 | 0.94 | [0.72, 0.99] |
| Age | 1.18 | [1.09, 1.39] | 1.09 | 0.84 | [0.72, 0.92] |
| Season | 1.18 | [1.08, 1.38] | 1.08 | 0.85 | [0.73, 0.92] |
| Others | 1.03 | [1.00, 1.84] | 1.02 | 0.97 | [0.54, 1.00] |

Figure S1: Diagnostic plot of the residuals of the zero-inflated Poisson GLMM.


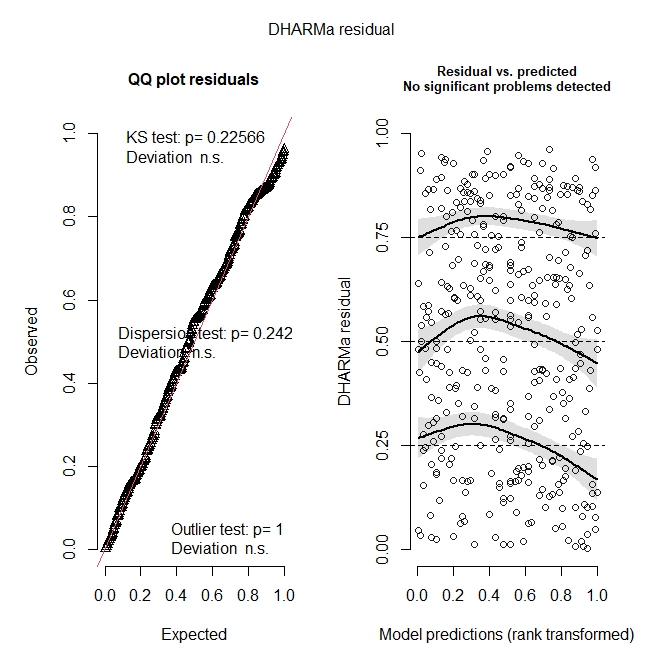


Figure S2: Diagnostic plot of the residuals of the zero-inflated Negative Binomial GLLVM.


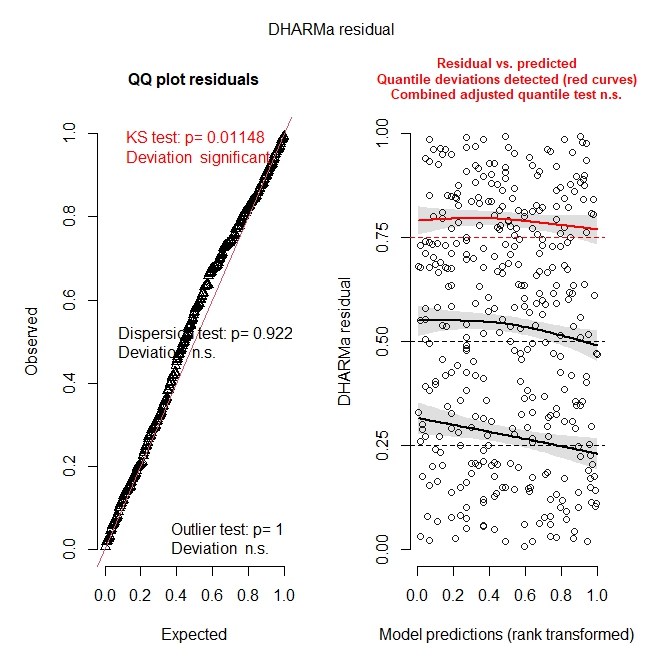


Figure S3: Diagnostic plot of the residuals of the zero-inflated Poisson GLLVM.


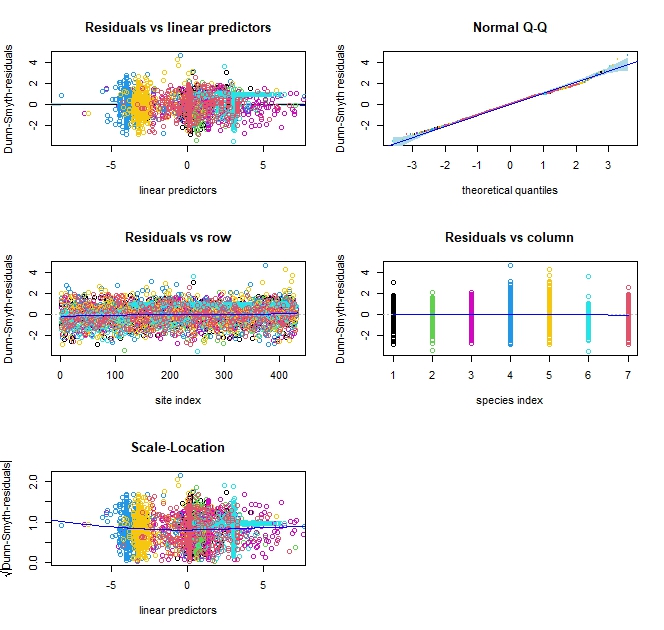

Supplement: Supplementary file 1 — Additional file 1: Table S1. Variation inflation factors (VIFs) with the zero-inflated Poisson GLMMs. Figure S1. Diagnostic plot of the residuals of the zero-inflated Poisson GLMM. Figure S2. Diagnostic plot of the residuals of the zero-inflated negative binomial GLLVM. Figure S3. Diagnostic plot of the residuals of the zero-inflated Poisson GLLVM. [file 13071_2023_5797_MOESM1_ESM.docx]
